# Supplementary material for: Deep-sequence phylogenetics to quantify patterns of HIV transmission in the context of a universal testing and treatment trial – BCPP/Ya Tsie trial
Source: eLife. 2022 Mar 1;11:e72657. doi: 10.7554/eLife.72657 (PMC8912920; doi:10.7554/eLife.72657)
Supplement: Supplementary file 1 [file elife-72657-supp2.zip › Supplementary file 2.odt]

Membership and affiliations of the Botswana Combination Prevention Project and PANGEA consortium

Botswana Combination Prevention Project:

Myron Essex1, Joseph Makhema1, Shahin Lockman1, Eric Tchetgen Tchetgen1, Molly Pretorius Holme1, Ingrid Bassett1, Kara Bennett1, Victor DeGruttola1, Scott Dryden-Peterson1, Kenneth Freedberg1, Vladimir Novitsky1, Kathleen Powis1, Roger Shapiro1, Rochelle Walensky1, Rui Wang1, Kathleen Wirth1, Marina Anderson2, Shenaaz El-Halabi2, Ernest B. Fetogang2, Refeletswe Lebelonyane2, Madisa Mine2, Tendani Gaolathe3, Simani Gaseitsiwe3, Poloko Kebaabetswe3, Mompati Mmalane3, Mosepele Mosepele3, Sikhulile Moyo3, Rosemary Musonda3, Erik van Widenfelt3, Pam Bachanas4, Ebi Bile4, Arielle Lasry4, Tafireyi Marukutira4, Lisa A. Mills4, Janet Moore4, Sherri Pals4, Elliot Raizes4, Chunfu Yang4 

Affiliations

1. Harvard T.H. Chan School of Public Health, Harvard University, Boston, MA

2. Ministry of Health, Republic of Botswana, Gaborone, Botswana

3. Botswana Harvard AIDS Institute Partnership, Gaborone, Botswana

4. Division of Global HIV/AIDS and TB, Centers for Disease Control and Prevention, Atlanta, Georgia, USA

PANGEA Consortium: 

Helen Ayles1, Rory Bowden2, Vincent Calvez3, Myron Cohen4, Tulio D'Oliveira5, Ann Dennis6, Max Essex7, Sarah Fidler8, Dan Frampton9, Richard Hayes10, Josh Herbeck11, Pontiano Kaleebu12, Cissy Kityo13, Jairam Lingappa14, Vladimir Novitsky15, Nick Paton16, Andrew Rambaut17, Janet Seeley18, Deogratius Ssemwanga19, Frank Tanser20

Affiliations

1. London School of Hygiene & Tropical Medicine, London, UK, 

2. University of Oxford, Oxford, UK 

3. Ecole Normale Supérieure de Lyon, Lyon, France 

4. University of North Carolina, Chapel Hill, NC, USA

5. University of KwaZulu-Natal, Durban, KwaZulu-Natal, South Africa 

6. University of North Carolina, Chapel Hill, NC, USA

7. Harvard University, Boston, MA, USA 

8. Imperial College London, London, UK

9. University College London, London, UK 

10. London School of Hygiene & Tropical Medicine, London, UK 

11. University of Washington, Seattle, WA, USA 

12. MRC/UVRI Uganda, Kampala, Uganda 

13. Joint Clinical Research Centre, Kampala, Uganda 

14. University of Washington, Seattle, WA, USA 

15. Africa Health Research Institute, Durban, KwaZulu-Natal, South Africa and University College London, London, UK

16. Yong Loo Lin School of Medicine, Singapore, Singapore

17. University of Edinburgh, Edinburgh, UK 

18. MRC/UVRI Uganda, Kampala, Uganda and London School of Hygiene & Tropical Medicine London, UK 

19. MRC/UVRI Uganda, Kampala, Uganda 

20. Africa Health Research Institute, Durban, KwaZulu-Natal, South Africa
